# The reasons of the nursing staff to notify adverse events^1^

**DOI:** 10.1590/0104-1169.3556.2476

**Published:** 2014

**Authors:** Miriam Cristina Marques da Silva de Paiva, Regina Célia Popim, Marta Maria Melleiro, Daisy Maria Rizatto Tronchim, Silvana Andréa Molina Lima, Carmen Maria Casquel Monti Juliani

**Affiliations:** 2 PhD, RN, Departamento de Enfermagem, Faculdade de Medicina de Botucatu, Universidade Estadual Paulista "Júlio de Mesquita Filho", Botucatu, SP, Brazil; 3 PhD, Assistant Professor, Departamento de Enfermagem, Faculdade de Medicina de Botucatu, Universidade Estadual Paulista "Júlio de Mesquita Filho", Botucatu, SP, Brazil; 4 PhD, Associate Professor, Escola de Enfermagem, Universidade de São Paulo, São Paulo, SP, Brazil

**Keywords:** Safety, Risk Management, Medical Errors, Nursing, Qualitative Research

## Abstract

**OBJECTIVE:**

this research aimed to understand the motivation for reporting adverse events from the perspective of nursing staff in the work environment.

**METHOD:**

qualitative study that used the phenomenology of Alfred Schutz for reference, which offers a systematic approach to understand the social aspects of human action. Data were collected by open interviews with 17 nurses and 14 technicians/assistant nurses in a university hospital.

**RESULTS:**

motivation was revealed through six categories: all types of occurrences must be reported; the incident report is an auxiliary instrument to health care provision management; the culture of punishment in transition; nurses as the agents responsible for voluntary reporting; sharing problems with higher management and achieving quality in the work process.

**DISCUSSION:**

it was unveiled that, when reporting adverse events, team members perceived themselves to be in a collaborative relationship with the institution and trusted that they would receive administrative support and professional security, which encouraged them to continue reporting. Reporting allows health care professionals to share responsibilities with managers and encourages corrective actions.

**FINAL CONSIDERATIONS:**

the study revealed the nursing staff's motivation for adverse event reporting, contributing to reflections on institutional policies aimed at patient safety in health care.

## Introduction

In the last few years, the voluntary reporting of adverse events has become an important instrument to improve quality in health care systems worldwide. The reporting system consists of interconnected actions aimed at detecting and analyzing adverse events (AE) and risk situations, so that professionals can learn from such events and improve patient safety during hospitalization^(^ 1 ^)^. However, studies show that, due to underreporting, this type of system does not capture the total number of AEs occurring in institutions^(^ 2 ^-^ 3 ^)^. Research in Brazil found that 76.8% of individuals never filled a notification and, internationally, over 40% of them never utilized this procedure and 25% did not know the reporting system^(^ 4 ^-^ 5 ^)^. Among the factors that interfere with underreporting are cultural and organizational aspects, practical health care structure, security systems and work regulations and processes^(^ 6 ^)^. The Hospital Survey on Patient Safety Culture indicates that the aspects that need to be improved in institutions, in relation to notification systems, are non-punitive responses to error and the number of reported events^(^ 7 ^)^.

In view of the statements above, the questions that prompted this study were: what motivates the nursing staff to report AEs and what has their experience with a reporting system for AEs implemented in the studied hospital been like? The objective of the investigation was formulated as understanding the reasons for AE reporting from the perspective of nursing professionals in the work environment.

The results of revealing these professionals motivation will certainly contribute to better understand the subject and may permit clarification and encourage reporting and support actions to settle the related negatives aspects and enhance the positives aspects, promoting patient safety in health care.

## Method

The choice of this reference is due to the relevance of Schutz' ideas to approach nursing actions in the reporting process of AE since, according to him, the situations in the world of daily life are shared and interpreted by the group, where each individual constructs his/her own view with contributions they are offered in their continuous interaction with their peers and based on an inventory of previous experiences, which operate as a reference code^(^ 8 ^)^. According to Schutz, this is the social context man lives in and relates to and, in accordance with the relationships and experiences, continues formatting his "biographic self", which distinguishes him from others, motivating him in his natural attitudes^(^ 8 ^)^. Social action, in turn, is practiced among two or more people. It is projected by man in a conscious and intentional way, and contains a subjective meaning that gives him the direction^(^ 8 ^)^.

In line with Schutz, "reasons to" instigate the accomplishment of the action and, therefore, are directed to the future. The "reasons why" are evident in the events already completed. They are the facts, are immutable, but not forgotten, and can influence the actions of the present^(^ 8 ^)^.

In this sense, social phenomenology seeks to learn about and organize what individuals experience in their daily lives, as elements that act, interact and complement one another, thus configuring a social group with typical characteristics. What matters in this study are the reasons that drive the action of these professionals, so they were heard on their experiences.

Study and data collection scenario: This study was developed at a university hospital in São Paulo state, Brazil, reference for more complex cases in the Unified Health Care System. The study received approval from the Research Ethics Committee of the Botucatu School of Medicine - UNESP, according to Official Letter 123/2011, dated April 04, 2011.

The criterion for inclusion in the study was being a Nurse or a Nursing Technician and/or Assistant Nurse, who had been working at the institution for longer than one year, with experience using the adverse-event reporting system, who expressed interest in participating in the study. All the participants signed an informed consent form.

Between June and July 2011, the research author interviewed 17 nurses and 14 technicians and/or assistant nurses, individually, with guarantee of privacy, anonymity and confidentiality of the information provided. At first, data to characterize the participants were collected. Then, two guiding questions were answered: 1. "Tell me what an AE is to you. Use examples if you want to." 2. "Tell me about your experience in relation to the occurrence of AEs and adverse-event reporting."

The total number of participants was not previously set. Instead, data collection was interrupted when data showed signs that the phenomenon had been unveiled, the researchers' concerns had been answered and the objectives achieved. The technicians and/or assistant nurses were interviewed after the nurses, since they use the AE reporting system less often. Interviews were identified with the letters "E" for nurses and TA for technicians and assistant nurses, followed by an ordinal number. The interviews, which lasted on average 10 minutes, were recorded and the tapes were destroyed after transcription of the content.

Data analysis: Units of meaning were obtained from the reading and description of the actions the subjects experienced and expressed in their testimonies, seeking what was common in the actions of professionals in reports of AE, the invariant. Then, the data were organized, searching for the typical action that was analyzed in the testimonies^(^ 8 ^)^. Finally, a comprehensive analysis of these groups was performed according to the motivational theory of A. Schutz^(^ 8 ^)^.

## Results

As the adopted reference, the contents of the statements were analyzed and the staff's motivation was represented in six categories: all types of occurrences must be reported; the incident report is an auxiliary instrument to health care provision management; the culture of punishment in transition; nurses as the agents responsible for voluntary reporting; sharing problems with higher management and achieving quality in the work process. These categories were grouped in ""reasons why" and "reasons for", following A. Schutz' motivation theory^(^ 8 ^)^. Among the categories that are related to "reasons why" to notify AEs, the professionals' statements define and exemplify the AEs that should be reported, composing the category: all types of occurrences must be reported. *(...)I understand that an AE refers to any event occurring to patients that escapes normality. For example: a patient's falling from the bed, a medication that is wrongly administered either by the wrong route or at a wrong dose, or it may be a transfusion reaction. It is anything unexpected that happens to the patient, anything that is not according to the institution's protocol and that may interfere and cause damage to patients (...) Even if it does not cause damage, but influences the work process both in nursing and in medical work *(E4.1,2). *(...) adverse events are those that occur without planning in the workplace* (E13.1).

In their discourse, the professionals express the understanding that the incident report is an auxiliary instrument to manage health care delivery, to identify the problem and seek alternatives to solve problems related to health care, whether in nursing or problems related to other areas. *(...) this adverse event report system has helped to identify the problems that have occurred in nursing care. It also serves as the reporting bulletin for other areas. It seeks to find solutions for this; it seeks to identify such problems and find a solution. To see another way or another method that is more suitable *(TA19.8). *(...) there are also the night staff members, who use the bulletin. Especially with regard to human resources deficit. They will do it if they have too many patients or the like* (E12.8).

Another category constructed refers to the culture of punishment in the institution:

*(...)At first, it was a problem, at first, when I had contact with it. I stayed away. When I arrived, it was already in use, and I was intimidated by the reporting bulletin, very intimidated. (...) then, it changed, (...) Today, reporting is natural* (E5.4). *(...) the team of assistant nurses and technicians, sometimes they feel a little afraid because we are writing this reporting bulletin. They know that they may be held accountable as well* (E16.7).

The last category, related to the "reasons why" of AEs reporting, is about the perception that nurses are primarily responsible for reporting, which is not mandatory: *(...)These events that occurred in the unit, I didn't report them myself, you know? I told the supervisor about them, and he completed the bulletin, the report, and sent it to the Nursing Division. Then, there was a reply, and everything worked out* (TA31.4).

The "reasons for" were constituted in two categories. The definitions emerged based on the analysis of excerpts from the statements that expressed the staff's motivation to notify the problems they experience in daily routine with top management. The confirmation of this sharing will be confirmed in the form of return as a result of notification of AE. *(...) my experiences have always been positive... I have always reported. The boss later gives me feedback about it, some written feedback is given, or if no further information needs to be added, she simply informs that she is aware of it... (...) I think it's a good channel* (E10.6,7). *(...) you attend to 11 children, sometimes. On some days, there are not enough employees. So, I think that reporting shows that you need support, that you are attending to this number of children, that they are aware of it, because if there are any problems, which may happen, you know, you're asking for support. (...) It's a way for you to explain, an explanation of something that happened too. (...) I think that it's a good idea to put it in writing. If there are any intercurrences, you have to put it in writing, to document it, to have institutional ... support* (TA18.3,4,7).

Another category that relates to expectations of nurses referred to the improvement of the work process. Professionals, who experience the AE, are motivated to notify to prevent its recurrence. *(...) we do have to notify and we will find out where the error lies, the problem, how to solve it, how to mitigate it. You have to search for the problem, I think, when it exists, you have to search. Found it? What we can do to prevent this from happening again?* (E4.10). *(...)Because we are humans, we try to prevent it, but we make mistakes, you know?! Because we are human beings. I think that we must try to prevent errors as hard as possible, but when they happen, these AE must be communicated. Because we have to improve, we have to correct our mistake* (TA30.4).

## Discussion

In this research, what was typical in the data analysis were the inconsistencies related to the patients' safety taxonomy, thus showing conflict with the classification of terms published by the World Health Organization (WHO)^(^ 9 ^)^. The team correctly recognized the term *adverse event* as an unexpected and undesired situation that brings damage to patients during health care provision. However, the team also incorporated definitions of *incident* and *harmless incident*, and brought other definitions that hinder comprehension and could cause confusion in the communication and information transfer that takes place in hospitals, such as the reporting instrument itself, during shift changes and patient transfers.

Although they show to understand the meaning of AE, the professionals reported the use of the reporting instrument to communicate different occurrences. They considered problem situations as the other occurrences reported, whether administrative, behavioral or organizational, which were related to various situations, such as conflicts, communication problems and redistribution of personnel and equipment. The types of conflicts occurring among team members, patients and their relatives and which are observed in the nursing team's everyday work are recorded in the statement. Communication failure certainly interferes with workers' satisfaction and work results. Pressured by problems in everyday work, nursing professionals use the reporting system to inform about their conflicts, and they seek help to arbitrate the situations.

It appears from the analysis that nursing professionals establishing their learning from their own experiences and those experienced with their peers, constituting, through intersubjectivity, according to Schutz, their baggage of knowledge^(^ 8 ^)^. Emphasizing the importance of patients and their relatives' participation for health care safety, it is useful to rethink existing institutional communication models and invest in the development of team members' communication skills. These may be factors that can promote efficient communication and create conditions to help professionals with the prevention of undesirable events^(^ 10 ^)^.

This study indicated that different professionals participate in the process of health care work and, hence, use the reporting system. In that way, as regards the reporting of adverse situations, the respondents perceived that health care is constructed by the development of interdependent processes, so that all the professionals involved become responsible for the results. It is observed that reporting establishes, among those who share the social reality, a relation in which intersubjectivity and intercommunication are present, since people live together, influence and understand one another, thus acting and receiving the actions from others^(^ 8 ^)^.

It is relevant to mention that nurses have been responsible for the organization and coordination of care provision activities in hospitals, as well as for making it possible for other professionals on the nursing team and others on the health care team to work in hospitals^(^ 11 ^)^. In this regard, the AE report presents itself as a data and information instrument that fosters communication among professionals and that is helpful to management.

Concerning management/staff dimensioning, the team's statements reveal the use of reporting to denounce the overload of activities with risk to patients. It is observed that, in situations where the team's capacity to provide care is disrespected, the pressure to do so forces professionals to rely on their memory more often to perform important actions, and this hinders effective communication among professionals, thus creating an environment of insecurity for care provision^(^ 12 ^)^.

Most of the professionals in this study reported that they believed in and disseminated the non-punitive purpose of AE reporting, thus showing the team's effort towards encouraging it and not relating it to unpleasant feelings and outcomes. However, they reveal fear in view of the investigation and analysis process and relate that accountability can be followed by orientation or warning.

Based on the biographical situation composed of prior knowledge and experience, conceptualized by Schutz^(^ 8 ^)^, the fear finds agreement in studies showing that health care professionals involved with errors suffer consequences that may have an administrative character, verbal and written punishment, dismissal as well as civil, legal and ethical procedures that may prevent their legal practice of the profession^(^ 13 ^)^. International organizations with the goal of monitoring and preventing errors instead of punishing professionals have repeatedly suggested that AE reporting should be encouraged as one of the main forms to access their real causes. The attempts to punish culprits have not reduced the frequency of AE and much less contributed to the creation of effective preventive strategies. They have an opposite effect as they induce under-reporting and hinder the implementation of protocols that can lead to error prevention^(^ 14 ^-^ 15 ^)^. In this study, reports of comprehensive conclusions of the analysis of the reported situations are predominant, as opposed to the fear of the notifications mentioned. In this perspective, it was revealed that the professionals were having positive experiences that led to the understanding of reporting as a tool aimed at improving the quality of care, beginning the deconstruction process of the characteristic types, relevant in social phenomenology^(^ 8 ^)^, and giving rise to transition situations of the institutional culture.

Although nursing technicians and assistant nurses understand that they can report, they show doubt concerning the authorization to do so, and they do not feel knowledgeable about how to record events. Hence, they prefer to report incidents to nurses so that they can later record it. In this way, nurses are appointed as the professionals responsible for AE reporting.

Nurses routinely play the role of nursing care supervisors due to their condition of team leaders. They are viewed as the ones who know about all the procedures associated with health care. Many times, in this activity, they emphasize work control and supervision and the recording of failures and sanctions. Nursing professionals and other team members acknowledge that nurses, in this condition, assume an authoritarian and centralizing attitude^(^ 16 ^)^.

Hence, the team's conceptualization concerning the nurse's role as the person responsible for reporting suggests a ranking of that action and makes it difficult for nursing technicians and assistant nurses, as well as for other professionals, to take responsibility for reporting the AE they experience. Furthermore, according to WHO, this conceptualization, in reality, needs to be reformulated so that frontline professionals can report undesirable facts, such as doctors, nurses, nursing technicians and auxiliaries, more than exclusively higher-ranked agents. Reporting systems must be designed to enable the processing of reports, including those from patients, their relatives and service users^(^ 10 ^)^. These individuals can contribute to the process by providing additional information about the events and subsequent impacts. Additionally, providing opportunities for all to report events promotes greater surveillance among health care service providers and organizations and permits integrating active participants in the search for improvement in patient safety^(^ 17 ^)^.

Underreporting may be related to the fact of it being restricted to records made by nurses and also to other causes, such as the voluntary, non-mandatory character, the lack of time and the habit to report. Integrated in the health care team and experiencing the environment of the reporting system, professionals intersubjectively share the need to define the role and should find conditions and have the freedom to choose and decide about the AE reporting action, contemplating their expectations of the social group as a participant in the daily experience^(^ 8 ^)^.

Regarding expectation when nursing professionals experience problems during their activities, they do not always feel secure or have the necessary resources to solve them. Hence, they seek support by sharing the problems with their bosses. The reason for these professionals to report is to establish a communication channel, and they expect to receive their bosses' opinions about their conduct, orientation about how to act or even the information that the problem will be solved at another instance.

Several characteristics of the nursing work environment directly affect the quality of the care given to patients. Structural failures and deficiency in work processes predispose to errors and cause work overload. Institutions that do not make efforts to promote good work conditions for the nursing staff may place their patients in a situation of greater vulnerability in relation to error occurrence^(^ 18 ^)^. In this regard, a study describes that the level of development of an organization, its work processes and professionals can directly affect care provision outcomes and observes that the majority of accidents and failures result from failures in the workplace's system^(^ 19 ^)^.

Regarding the reporter's relationship with managers, the nursing professionals' statements showed the perception of the importance of managers' opinions recorded in the feedback to reporters. Such feedback establishes the intercommunication that grants meaning to the motivated action^(^ 8 ^)^. When they participate in the reporting system, nursing professionals perceive themselves as contributors to the institution. However, the time elapsed until the reports are returned or the lack of knowledge about the effect produced on managers can indicate that intercommunication did not occur, and that produces dissatisfaction and demotivation due to the lack of recognition of the collaboration provided.

Stressful situations are frequent in nursing professionals' lives, particularly in institutions with scarce resources, as often is the case of public hospitals. Responsible for providing specialized care as well as the material and technological resources - process and structure - necessary for patient care, those professionals are pressured to make decisions, which many times pose risk to patients' and their own integrity^(^ 20 ^)^. The situations reported in this study show that the team's reasons for reporting are to have administrative support and institutional security when situations of tension and risk are communicated. This encourages them to report the difficulties and anguish that are frequently experienced in the care practice. These situations from the everyday world, shared and interpreted by the group, involving people, transform the conduct in social action and, according to Schutz, characterize it as typical^(^ 8 ^)^.

It is recommended that managers of health care organizations, in agreement with their employees, should define and document policies for managing risk situations. Risks must be identified and analyzed according to their origins and, based on such diagnosis, preventive actions must be implemented^(^ 21 ^)^. In order to contribute to the identification of risk situations and their management, the AE reporting bulletin, as an institutional document, must be preferably anonymous and confidential, and should not be used as an instrument to accuse professionals^(^ 22 ^)^.

In this study, the contribution to the prevention of future events, the learning obtained from the investigations and the non-punitive effect of reporting are perceived as a benefit of the system, expectation that provides security to professionals.

The statements in this study showed the team's motivation to report in order to correct and improve work processes continuously, so as to prevent future AE and prevent damage to patients. In the context of quality, the meaning of continuous improvement is the incessant search for error elimination as a way to adequately qualify the outcomes^(^ 23 ^)^. In order to solve quality problems, the first step is to examine each phase of the process so as to prevent problems before they occur, instead of correcting them after they have happened. However, despite providing visibility to failures, only reporting is not enough to achieve continuous improvement. To implement the continuous improvement of processes, it is necessary to use an established, tested and reliable methodology that is supported by effective instruments and permits the achievement of preset objectives. The systematic and problem-focused approach allows for the identification of problem causes and the development and implementation of solutions and action plans for process improvement^(^ 24 ^)^.

## Final Considerations

The approach used in developing this study enabled us to understand the perception of nursing professionals that the AE reporting system helps patient care management, that it allows health care professionals to share responsibilities with managers and encourages corrective actions, aimed at not repeating errors and at preventing future AE. It showed to be useful in order to denounce the inadequacy of human resources, as well as other fragilities of the institution. The expectation of administrative support and professional security is unveiled, as these conditions encourage professional to report the difficulty and anguish, experienced in the health care provision practice. When professionals decide to report AE, their motivation is to cooperate with the institution, and they expect to receive feedback in the form of help to review the conducts taken and to achieve higher problem-solving levels. The knowledge that emerged from the professionals' experiences point to the need to disseminate the WHO taxonomy in patient safety in order to improve information quality and encourage reporting. The typical aspect in this study is verbalized by disseminating the non-punitive purpose of AE reporting, thus showing the existence of an effort to encourage reporting and not relating it to unpleasant feelings and consequences. The respondents' view of nurses as the professionals responsible for AE reporting contributes to make it difficult for assistant nurses and technicians as well as for other professionals to consistently commit themselves in that sense. It is necessary to demystify nurse-centered reporting and to promote opportunities for orientation, clarification and encouragement towards participation by all professionals.

This study shows the importance of understanding the subjective aspects of nursing professionals' action in the AE reporting system, through the knowledge about the expectations and reasons that permeate their decisions and conducts. This research is limited by the fact that the interviewed group contained more nurses and that, as a phenomenological study, the results are not generalizable to other populations. Nevertheless, the understanding provided by the framework of Alfred Schutz can contribute to reflections on the institutional policy and the improvement of work processes, aiming to enhance the safety of care.
